# Convalescent plasma donors show enhanced cross‐reactive neutralizing antibody response to antigenic variants of SARS‐CoV‐2 following immunization

**DOI:** 10.1111/trf.16934

**Published:** 2022-06-02

**Authors:** Heli Harvala, Dung Nguyen, Peter Simmonds, Abigail A. Lamikanra, Hoi Pat Tsang, Ashley Otter, Piet Maes, Mhairi Webster, Adam Clarkson, Fotini Kaloyirou, Valerie Hopkins, Stephen M. Laidlaw, Miles Carroll, Ana Mora, Alexandra Griffiths, Sheila MacLennan, Lise Estcourt, David J. Roberts

**Affiliations:** ^1^ Microbiology Services NHS Blood and Transplant Colindale UK; ^2^ Infection and Immunity Univeristy College of London London UK; ^3^ Nuffield Department of Medicine, Peter Medawar Building for Pathogen Research University of Oxford Oxford UK; ^4^ Wellcome Centre for Human Genetics, Nuffield Department of Medicine, Roosevelt Drive, Headington University of Oxford Oxford UK; ^5^ Clinical Services NHS Blood and Transplant Oxford UK; ^6^ UK Health Security Agency Porton Down, Salisbury UK; ^7^ Clinical and Epidemiological Virology KU Leuven, Rega Institute Leuven Belgium; ^8^ Statistics and Clinical Research NHS Blood and Transplant Cambridge UK; ^9^ Statistics and Clinical Studies NHS Blood and Transplant Bristol UK; ^10^ Clinical Services NHS Blood and Transplant Barnsley UK; ^11^ Radcliffe Department of Medicine and BRC Haematology Theme University of Oxford Oxford UK

**Keywords:** antibody neutralization, antigenic variants, convalescent plasma, COVID‐19, Delta, Omicron, SARS‐CoV‐2, vaccination

## Abstract

**Background:**

The therapeutic benefit of convalescent plasma (CP) therapy to treat COVID‐19 may derive from neutralizing antibodies (nAbs) to SARS‐CoV‐2. To investigate the effects of antigenic variation on neutralization potency of CP, we compared nAb titers against prototype and recently emerging strains of SARS‐CoV‐2, including Delta and Omicron, in CP donors previously infected with SARS‐CoV‐2 before and after immunization.

**Methods and Materials:**

Samples were assayed from previously SARS‐CoV‐2 infected donors before (*n* = 17) and after one (*n* = 43) or two (*n* = 71) doses of Astra‐Zeneca or Pfizer vaccinations. Ab titers against Wuhan/wild type (WT), Alpha, Beta, and Delta SARS‐CoV‐2 strains were determined by live virus microneutralization assay while titers to Omicron used a focus reduction neutralization test. Anti‐spike antibody was assayed by Elecsys anti‐SARS‐CoV‐2 quantitative spike assay (Roche).

**Results:**

Unvaccinated donors showed a geometric mean titer (GMT) of 148 against WT, 80 against Alpha but mostly failed to neutralize Beta, Delta, and Omicron strains. Contrastingly, high GMTs were observed in vaccinated donors against all SARS‐CoV‐2 strains after one vaccine dose (WT:703; Alpha:692; Beta:187; Delta:215; Omicron:434). By ROC analysis, reactivity in the Roche quantitative Elecsys spike assay of 20,000 U/mL was highly predictive of donations with nAb titers of ≥1:640 against Delta (90% sensitivity; 97% specificity) and ≥1:320 against Omicron (89% sensitivity; 81% specificity).

**Discussion:**

Vaccination of previously infected CP donors induced high levels of broadly neutralizing antibodies against circulating antigenic variants of SARS‐CoV‐2. High titer donations could be reliably identified by automated quantitative anti‐spike antibody assay, enabling large‐scale preselection of high‐titer convalescent plasma.

**Abbreviations**
CP: convalescent plasma
COVID‐19: coronavirus disease 2019
SARS‐CoV‐2: Severe acute respiratoty syndrome coronavirus 2
nAbs: neutralising antibodies
WT: wild type
GMT: geometric mean titre
MNA: microneutralization assay
FRNT: focus reduction neutralization test

## 1 INTRODUCTION

During the pandemic, SARS‐CoV‐2 has continued to rapidly evolve to evade immune responses, with many variants displaying multiple mutations in the spike gene that have been shown to reduce their susceptibility to neutralizing antibodies.^1^ These antigenic changes potentially contribute to immune evasion^2^ and may abrogate neutralization by monoclonal antibodies (mAbs) recently deployed as immunotherapy.^3^, ^4^, ^5^ Convalescent plasma has been shown to be an effective and affordable treatment if given soon after infection or in immunocompromised patients,^6^, ^7^ although its efficacy may be conditioned by the ability of anti‐SARS‐CoV‐2 antibodies to effectively neutralize currently circulating strains.^8^, ^9^ We investigated whether individuals with previous SARS‐CoV‐2 infection who have been subsequently immunized represent an effective source of high‐titer cross‐reactive nAb for immunotherapy. Such plasma is urgently needed to assess its treatment efficacy for immunocompromised individuals infected with recently emerging Delta and Omicron antigenic variants of SARS‐CoV‐2.

## 2 METHODS

### 2.1 Convalescent plasma collections in England

A cohort of registered NHS Blood and Transplant (NHSBT) convalescent donors with a suspected or laboratory confirmed SARS‐CoV‐2 infection and subsequently known to be immunized were invited to the current study (Table S1). SARS‐CoV‐2 type was inferred based on their first convalescent plasma donation date—WT: April–December 2020; Alpha: January–March 2021 (based on https://www.gisaid.org). We targeted donors who had evidence of a moderate level of anti‐SARS‐CoV‐2 antibodies prior to immunization (see below).^10^ A patient information leaflet was given for those interested to join this study and an appointment for blood sample in the NHSBT Birmingham donor center was provided after signed consent was received. Up to two blood samples were collected a minimum of 28 days after the immunization from the individuals who had previously donated COVID‐19 convalescent plasma. An archive sample of their previous donation given prior to vaccination was also obtained when available.

### 2.2 Ethical approval

Approval for this study was received from the West Midlands Solihull Research Ethics Committee, UK (REC reference: 21/WM/0082, IRAS project ID: 296926).

### 2.3 Study participants

A total of 131 samples were obtained from 94 convalescent donors originally infected with SARS‐CoV‐2; 80 of them had moderate anti‐SARS‐CoV‐2 antibody levels in the IgG EUROimmun assay targeting the spike S1 domain (Perkin Elmer; S/Co ratio 1–5.99) and 14 with higher antibody levels (S/Co ratio > 5.99) prior to immunization. A total of 17 convalescent samples were taken from donors prior to their immunization, and 114 samples taken from donors after receiving one or two doses of AstraZeneca or Pfizer vaccines (43 samples taken 33 to 79 days after the first dose and 71 samples 29 to 140 days after the second dose). Most donors received AstraZeneca vaccine (87/94, 93%), while the remainder were immunized with the Pfizer vaccine (both vaccines based on WT sequence). All three samples were available for five donors whereas two samples were available for 27 donors and one post‐immunization sample for 62 donors, from which most provided sample after second dose of vaccine (*n* = 49). All samples were taken before the third booster dose was introduced. Donors were aged between 21 and 65 years (mean age 50 years), and most were males (75/94, 80%).

### 2.4 Detection of neutralizing antibodies

The presence of SARS‐CoV‐2 nAbs in plasma samples were determined using a live virus microneutralization assay (MNA) with WT (England‐2), Alpha (B.1.1.7), Beta (B.1.351), and Delta (B.1.617.2) strains as previously described.^8^ A selection of samples (*n* = 33) were tested for nAb to Omicron (B.1.1.529) using focus reduction neutralization test (FRNT). This was necessitated by the absence of cytopathology in cells infected with Omicron, preventing the use of a traditional MNA.^11^ However, to ensure nAb titers produced by the two assays were comparable, we assayed 33 samples in MNA and FRNT assays against WT SARS‐CoV‐2 and calibrated results by a regression analysis of nAb titers (Figure S2). These samples were selected to provide a mixture of samples obtained pre‐ and post‐vaccine and a range of Nab titers. Nab titers were comparable in two assay formats, necessitating only minor correction using the formula MNA = (FRNT – 0.159)/1.071 when comparing assay results.

All samples were assayed by Elecsys anti‐SARS‐CoV‐2 quantitative spike assay using a WT receptor binding domain recombinant protein as antigen (Roche, London, UK).

Statistical analysis was performed using the SPSS software, version 28.

## 3 RESULTS

### 3.1 Neutralizing antibody response after natural infection and vaccination

**FIGURE 1:**
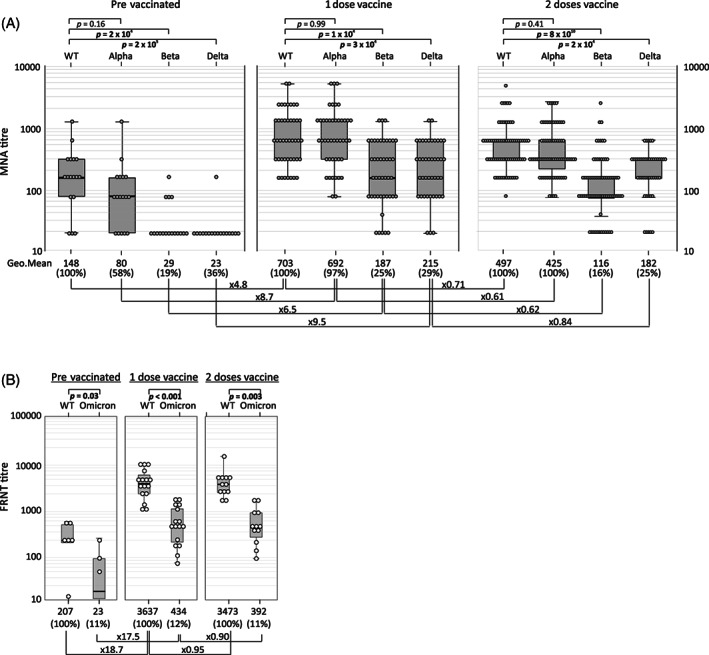
Comparison of neutralizing antibody titers against each SARS‐CoV‐2 strain in plasma from infected, prevaccinated individuals with those receiving 1 or 2 doses of vaccine obtained by microneutralization assay (A) and focus reduction neutralization assay (B). Median values of reactivity and fold change from reactivity to the WT strain are shown under each graph. Further comparisons of fold‐changes in reactivity after immunization with 1 or 2 vaccine doses are shown along links. Statistical comparisons of antibody levels induced by different SARS‐CoV‐2 strains used the Spearman rank correlation test (*p* values <.05 shown in bold).

A total of 131 samples were obtained from 94 convalescent donors originally infected most likely with either WT (*n* = 74) or Alpha variants of SARS‐CoV‐2 (*n* = 20). Neutralizing antibody against WT (geometric mean titer [GMT] 1:148, range <1:20–1:1280) and Alpha (GMT 1:80, <1:20–1:1280) variants were significantly higher than to Beta (GMT 1:29, <1:20–1:160) and Delta (GMT 1:23, <1:20–1:160) variants (*n* = 17, *p* < .001, Figure 1A). Neutralizing antibodies quantified by FRNT against WT (GMT 1:207, <1:20–1:534) were significantly higher than those measured against Omicron (GMT 1:23, <1:20–1:230) (*n* = 6, *p* < .001, Figure 1B).

**FIGURE 2:**
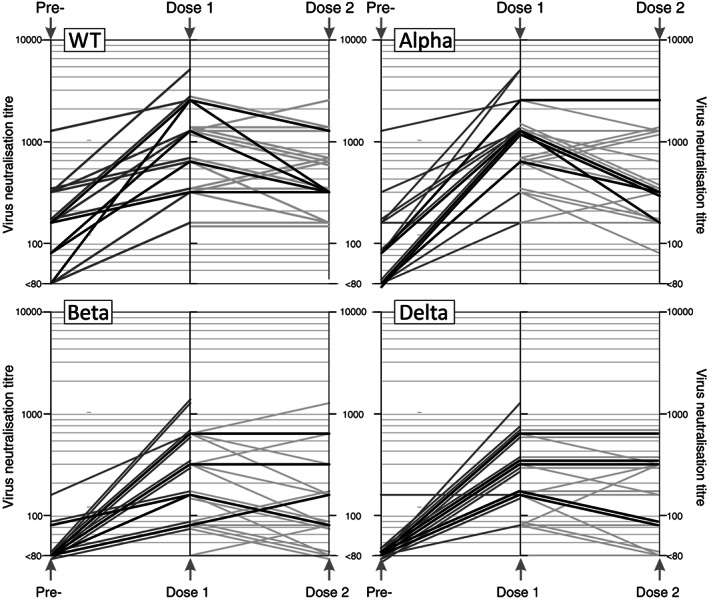
Time course of neutralizing antibody levels to each SARS‐CoV‐2 strain in sequential samples from pre‐ and post‐vaccinated subjects (time intervals and totals listed in Table S1); gray lines indicate results from subjects with samples collected pre‐ and post‐single vaccine does, or between dose 1 and dose 2 vaccinations.

The largest increase in the nAb titers were seen after first dose of vaccine (WT 4.8‐fold; Alpha 8.7‐fold; Beta 6.5‐fold and Delta 9.5‐fold increase in MNA; *n* = 43, Figure 1A); levels in sequential samples are shown in Figure 2). Similarly, 17.5‐fold and 18.9‐fold increases following immunization were observed against WT and Omicron strains respectively in the FRNT (Figure 1B). Actual nAb levels varied, with highest levels measured after first dose of vaccine against WT or Alpha with GMTs of 1:703 (1:160–1:5120) and 1:692 (1:80–1:5120) respectively compared to GMTs of 1:215 (<1:20–1280) against Delta and 1:187 (<1:20–1280) against Beta. The FRNT titers measured against the Omicron were similarly reduced when compared to WT(GMT 1:434 and 1:3637, respectively). Titers did not change significantly after the second dose of vaccine (Figures 1 and 2). No significant differences in neutralizing antibody titers were seen between donors who received Astra Zeneca or Pfizer vaccine (data not shown).

### 3.2 Predicting samples most suitable for convalescent plasma therapy using binding antibody titers

**FIGURE 3:**
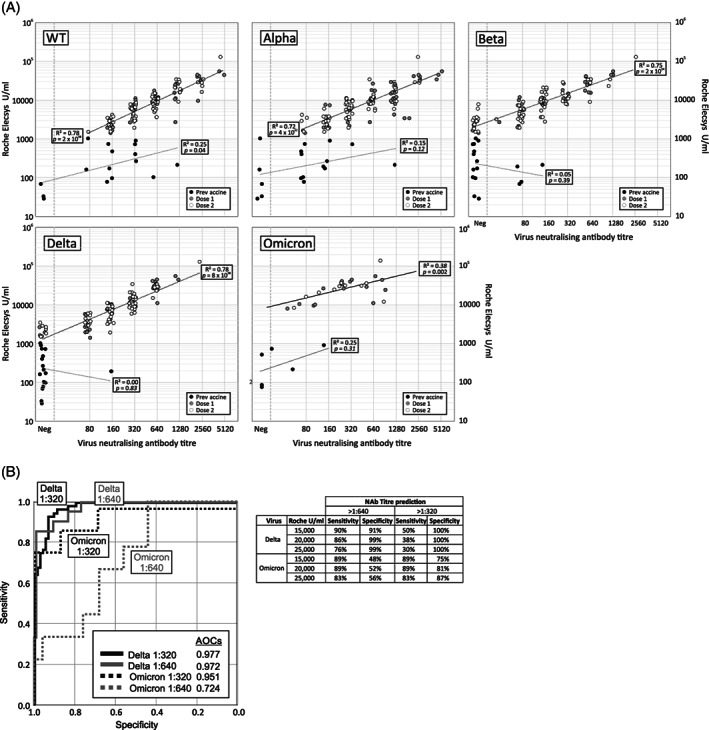
(A) Associations between neutralizing antibody titers to each SARS‐CoV‐2 strain by microneutralization assay (*x*‐axis panels) and in the Roche Elecsys assay (*y*‐axis). Samples collected prevaccination and after 1 or 2 vaccination doses are plotted separately (see key). Neutralizing antibody titer values have been jittered by ±1.2 fold to avoid overlapping points. Datapoints lines of best fit of log transformed values from both assays were separately plotted for samples collected pre‐ and post‐vaccination; *R* ^2^ and *p* values shown. (B) Receiver operating characteristic (ROC) analysis to evaluate the predictive value of the Roche S quantitative assay for neutralizing antibody titers of 1:640 and 1:320 against Delta and Omicron. The sensitivity and specificity of chosen Roche antibody levels to predict high titer convalescent donations containing a minimum neutralizing antibody titer of 1:640 or 1:320 against Delta and Omicron variants is tabulated to the right.

Virus neutralization titers and reactivity in Roche Elecsys spike quantitative antibody assays were compared using a total of 131 samples obtained from convalescent plasma donors (Figure 3A; Roche result was not available for three samples). Antibody titers measured by the Roche Elecsys spike total antibody assay were significantly associated with nAb titers against each SARS‐CoV‐2 strain in samples obtained from vaccinated donors; this was evident also with Omicron despite testing of smaller number of samples. However, the lower levels of nAbs detected in samples from prevaccinated donors showed little (WT; *p* = .04) to no (Alpha, Beta, Delta, and Omicron) significant association with reactivity in the Elecsys assay. Furthermore, infection induced relatively weaker reactivity in the Elecsys spike assay than would be predicted from neutralizing titers. Vaccination appears therefore to induce a much greater proportion of immunoluminescence‐detected antigen‐reactive antibodies and a qualitatively different antibody response.

A neutralizing antibody titer of 1:640 against Delta was selected as a likely therapeutic threshold for the use of convalescent plasma in patients infected with this strain, or possible subsequent variants. That level of nAbs should allow a titer of more than 1:100 to be achieved in an average recipient, based on the dilution of 500–560 ml of CP (2 × 250–280 ml plasma donations) into a plasma volume of around 2.5–3 liters in the recipient. These levels have been shown to protect also against reinfection in a non‐human primate model for SARS‐CoV‐2.^12^ The optimal cut‐off value in the Roche Elecsys assay in terms of specificity and sensitivity for predicting samples with ≥1:640 nAb titers to be used for plasma selection was determined by receiver operating characteristic (ROC) analysis (21 samples with titers ≥1:640; 110 samples <1:640). Antibody titers between 10,000 and 35,000 U/mL obtained by the Roche assay were selected as potential cut‐off values for sensitivity and specificity analysis (Figure 3B); a titer of 20,000 U/mL correctly identified 88% of donations (19/21) above 1:640, whereas 97% of donations below this nAb threshold were classified correctly as below 1:640 (107/110). A further analysis of the Roche Elecsys assay's ability to predict donations with a normalized nAb levels of >1:640 and >1:320 against Omicron were similarly evaluated by ROC analysis. While the precision of this analysis would benefit from larger numbers, a level of 20,000 U/mL in the Roche assay in those with previous infection followed by vaccination shows a sensitivity of 89% and specificity of 81% for predicting units with titers >1:320.

Based on these calculations, and in the absence of scalable nAb test for donation screening, we propose that convalescent plasma donations could be selected with a minimum antibody level in the Roche Elecsys spike total assay.

## 4 DISCUSSION

In England, NHSBT collected convalescent plasma from individuals with confirmed or suspected SARS‐CoV‐2 infection at least 28 days after the resolution of their symptoms between 22 April 2020 and 18 March 2021. Donations containing a minimum nAb titer of 1:100 were provided for two clinical trials based on Euroimmun IgG testing but collections were stopped as the analysis of trial results did not show overall benefit for hospitalized patients^13^, ^14^ However, the results were suggestive of possible benefit in the immunocompromised patient group inviting further trials with high‐titer plasma in this particular subgroup.

The study findings demonstrate that vaccinated convalescent donors develop high levels of cross‐reactive neutralizing antibodies against WT and newly emerging virus variants. This is important as new SARS‐CoV‐2 variants continue to emerge and may lead to reduction in the neutralization capacity of collected convalescent plasma. In the study sample of 94 convalescent plasma donors initially infected with either WT or Alpha variant, 21 also developed high levels of nAb against Beta, Delta and Omicron variants after the first vaccine dose. This data is consistent with other recently published evidence indicating that a single dose of mRNA SARS‐CoV‐2 vaccine in individuals who have had a previous natural infection will indeed elicit higher antibody titers than measured in vaccinated individuals who have not been infected by SARS‐CoV‐2.^15^, ^16^, ^17^ Recently published studies show that a single dose of mRNA vaccine boosts preexisting immunity of individuals toward new SARS‐CoV‐2 variants, including Omicron, that they have not been previously infected with.^17^, ^18^, ^19^

Plasma from vaccinated convalescent donors produce high levels of cross‐reactive nAb against newly emerging variants; these have potential therapeutic value for COVID‐19 and their polyclonal nature may be advantageous compared to monoclonal antibodies therapies that have substantially lost their neutralization capacity against antigenic variants of SARS‐CoV‐2. While the FRNT had to be used to quantify nAbs to Omicron, MNA and FRNT assays determined comparable titers for WT virus (Figure S1), although it is possible in principle that this relationship may differ for other SARS‐CoV‐2 strains. Further cross‐antibody comparisons will be required to address this conclusively.

Finally, we have shown that these post‐vaccinated high‐titer donors with cross‐reactive antibodies can be readily identified by automated quantitative spike assay; this provides the means for scalable, rapid, and large‐scale prospective collection of high titer donations against SARS‐CoV‐2 strains circulating to date from previously infected and subsequently vaccinated individuals.

## FUNDING INFORMATION

The study was funded by the European Commission (HORIZON2020 project Support‐E, no. 101015756) to HH, LE, and DJR. LE and DJR were also supported by the NIHR plasma grant (RECPLAS) and NHS Blood and Transplant R&D funding. DN and SL were supported by the Oak Foundation grant of MC.

## CONFLICT OF INTEREST

The authors have disclosed no conflicts of interest.

## Supporting information


**Appendix S1** Supporting Information
