# Supplementary material for: Convalescent plasma donors show enhanced cross‐reactive neutralizing antibody response to antigenic variants of SARS‐CoV‐2 following immunization
Source: Transfusion. 2022 Jun 2;62(7):1347–54. doi: 10.1111/trf.16934 (PMC9348319; doi:10.1111/trf.16934)
Supplement: Supplementary file 1 — Appendix S1 Supporting Information [file TRF-62-1347-s001.docx]

**Supplementary Data**

**Table S1**

Donation dates and vaccination intervals (days) for the study subjects.

| **Donor** | **Date of first CP donation** | **Likely infecting SARS-CoV-2 type** | **Time from first CP donation to 1st dose of vaccine** | **Time from 1st dose of vaccine to sample** | **Time from 2nd dose of vaccine to sample** | **Type of vaccine recieved** |
| --- | --- | --- | --- | --- | --- | --- |
| *All three samples obtained (pre-vaccine, post-dose 1 and post-dose 2) from 5 donors:* | | | | | | |
| **1** | 27/11/2020 | WT | 77 | 73 | 73 | AstraZeneca |
| **2** | 31/08/2020 | WT | 193 | 50 | 75 | AstraZeneca |
| **3** | 05/06/2020 | WT | 293 | 48 | 46 | AstraZeneca |
| **4** | 24/06/2020 | WT | 256 | 73 | 36 | AstraZeneca |
| **5** | 11/06/2020 | WT | 274 | 70 | 54 | AstraZeneca |
| *Two samples obtained (pre-vaccine and post-dose 1 or post-dose 2) from 12 donors:* | | | | | | |
| **6** | 24/11/2020 | WT | 112 | 43 | *NA* | AstraZeneca |
| **7** | 06/11/2020 | WT | 102 | 71 | *NA* | Pfizer |
| **8** | 11/08/2020 | WT | 191 | 72 | *NA* | AstraZeneca |
| **9** | 16/06/2020 | WT | 273 | 50 | *NA* | AstraZeneca |
| **10** | 21/12/2020 | WT | 70 | 65 | *NA* | AstraZeneca |
| **11** | 01/07/2020 | WT | 266 | 37 | *NA* | AstraZeneca |
| **12** | 03/06/2020 | WT | 288 | 62 | *NA* | AstraZeneca |
| **13** | 12/06/2020 | WT | 280 | 61 | *NA* | AstraZeneca |
| **14** | 05/02/2021 | Alpha | 26 | 70 | *NA* | AstraZeneca |
| **15** | 06/11/2020 | WT | 135 | 48 | *NA* | AstraZeneca |
| **16** | 21/08/2020 | WT | 180 | *NA* | 29 | Pfizer |
| **17** | 30/05/2020 | WT | 153 | *NA* | 12 | Pfizer |
| *Two samples obtained (post-dose 1 and post-dose 2) from 15 donors:* | | | | | |  |
| **18** | 04/11/2020 | WT | 136 | 39 | 69 | AstraZeneca |
| **19** | 26/01/2021 | Alpha | 35 | 55 | 48 | AstraZeneca |
| **20** | 07/10/2020 | WT | 155 | 46 | 69 | AstraZeneca |
| **21** | 23/11/2020 | WT | 117 | 37 | 41 | AstraZeneca |
| **22** | 09/12/2020 | WT | 94 | 49 | 36 | AstraZeneca |
| **23** | 22/09/2020 | WT | 177 | 50 | 72 | AstraZeneca |
| **24** | 22/12/2020 | WT | 74 | 62 | 56 | Pfizer |
| **25** | 06/06/2020 | WT | 294 | 42 | 56 | AstraZeneca |
| **26** | 02/03/2021 | Alpha | 26 | 38 | 39 | AstraZeneca |
| **27** | 04/12/2020 | WT | 95 | 52 | 57 | AstraZeneca |
| **28** | 30/12/2020 | WT | 88 | 33 | 49 | AstraZeneca |
| **29** | 26/06/2020 | WT | 256 | 66 | 71 | AstraZeneca |
| **30** | 20/02/2021 | Alpha | 35 | 44 | 36 | AstraZeneca |
| **31** | 26/08/2020 | WT | 201 | 56 | 68 | AstraZeneca |
| **32** | 28/12/2020 | WT | 52 | 79 | 88 | Pfizer |
|  |  |  | **Mean** | **55** | **56** |  |
|  |  |  |  |  |  |  |
| *One sample obtained (post-dose 1 or post-dose 2) from 62 donors:* | | | | | |  |
| **33** | 21/11/2020 | WT | 101 | 57 | NA | AstraZeneca |
| **34** | 15/01/2021 | WT or Alpha | 50 | 53 | NA | AstraZeneca |
| **35** | 08/06/2020 | WT | 255 | 78 | NA | Pfizer |
| **36** | 07/11/2020 | WT | 107 | 70 | NA | Pfizer |
| **37** | 09/01/2021 | WT or Alpha | 66 | 59 | NA | AstraZeneca |
| **38** | 22/02/2021 | Alpha | 32 | 50 | NA | AstraZeneca |
| **39** | 17/02/2021 | Alpha | Unknown | NA | 57 | Pfizer |
| **40** | 16/07/2020 | WT | 198 | NA | 46 | Pfizer |
| **41** | 04/07/2020 | WT | 200 | NA | 31 | Pfizer |
| **42** | 14/08/2020 | WT | 141 | NA | 54 | Pfizer |
| **43** | 12/02/2021 | Alpha | 20 | 65 | NA | AstraZeneca |
| **44** | 05/05/2020 | WT | 314 | NA | 46 | AstraZeneca |
| **45** | 10/08/2020 | WT | 162 | NA | 78 | AstraZeneca |
| **46** | 06/08/2020 | WT | 136 | NA | 118 | Pfizer |
| **47** | 09/01/2021 | WT or Alpha | 54 | 65 | NA | AstraZeneca |
| **48** | 06/03/2021 | Alpha | Unknown | 67 | NA | AstraZeneca |
| **49** | 20/05/2020 | WT | 229 | NA | 112 | Pfizer |
| **50** | 09/12/2020 | WT | 138 | 72 | NA | AstraZeneca |
| **51** | 20/07/2020 | WT | 280 | 74 | NA | AstraZeneca |
| **52** | 27/11/2020 | WT | 105 | NA | 43 | AstraZeneca |
| **53** | 03/10/2020 | WT | 165 | NA | 39 | AstraZeneca |
| **54** | 04/05/2020 | WT | 308 | NA | 45 | AstraZeneca |
| **55** | 21/09/2020 | WT | Unknown | NA | 45 | AstraZeneca |
| **56** | 31/07/2020 | WT | Unknown | NA | 33 | Not known |
| **57** | 23/06/2020 | WT | 278 | NA | 62 | AstraZeneca |
| **58** | 01/02/2021 | Alpha | 28 | NA | 57 | AstraZeneca |
| **59** | 17/09/2020 | WT | 162 | NA | 63 | Pfizer |
| **60** | 23/12/2020 | WT | 68 | NA | 52 | AstraZeneca |
| **61** | 06/07/2020 | WT | Unknown | NA | 102 | AstraZeneca |
| **62** | 14/11/2020 | WT | 65 | NA | 100 | Pfizer |
| **63** | 12/02/2021 | Alpha | Unknown | NA | 63 | AstraZeneca |
| **64** | 12/06/2020 | WT | 202 | NA | 126 | Pfizer |
| **65** | 18/06/2020 | WT | 303 | NA | 29 | AstraZeneca |
| **66** | 01/06/2020 | WT | 318 | NA | 34 | AstraZeneca |
| **67** | 08/06/2020 | WT | 266 | NA | 47 | AstraZeneca |
| **68** | 30/11/2020 | WT | 42 | NA | 117 | Pfizer |
| **69** | 13/06/2020 | WT | 308 | NA | 39 | AstraZeneca |
| **70** | 03/07/2020 | WT | 253 | NA | 55 | AstraZeneca |
| **71** | 15/08/2020 | WT | 245 | NA | 31 | AstraZeneca |
| **72** | 27/01/2021 | WT or Alpha | 134 | 44 | NA | Pfizer |
| **73** | 29/12/2020 | WT | 84 | NA | 40 | AstraZeneca |
| **74** | 23/01/2021 | WT or Alpha | 53 | NA | 39 | AstraZeneca |
| **75** | 18/07/2020 | WT | 244 | NA | 63 | AstraZeneca |
| **76** | 24/07/2020 | WT | 145 | NA | 140 | Pfizer |
| **77** | 24/02/2021 | Alpha | 107 | 47 | NA | Pfizer |
| **78** | 08/03/2021 | Alpha | 38 | NA | 34 | AstraZeneca |
| **79** | 06/08/2020 | WT | 156 | NA | 133 | Pfizer |
| **80** | 10/02/2021 | Alpha | 12 | NA | 78 | AstraZeneca |
| **81** | 30/09/2020 | WT | 101 | NA | 119 | Pfizer |
| **82** | 11/01/2021 | WT or Alpha | 71 | NA | 57 | AstraZeneca |
| **83** | 07/12/2020 | WT | Unknown | NA | 55 | Not known |
| **84** | 16/06/2020 | WT | 298 | NA | 55 | AstraZeneca |
| **85** | 15/06/2020 | WT | 284 | NA | 31 | AstraZeneca |
| **86** | 18/06/2020 | WT | 258 | NA | 74 | AstraZeneca |
| **87** | 06/07/2020 | WT | 288 | NA | 44 | AstraZeneca |
| **88** | 02/09/2020 | WT | 152 | NA | 102 | Pfizer |
| **89** | 27/06/2020 | WT | 202 | NA | 119 | Pfizer |
| **90** | 29/01/2021 | WT or Alpha | 26 | NA | 93 | Pfizer |
| **91** | 11/08/2020 | WT | 270 | NA | 39 | AstraZeneca |
| **92** | 27/05/2020 | WT | 249 | NA | 115 | AstraZeneca |
| **93** | 19/05/2020 | WT | Unknown | NA | 133 | Pfizer |
| **94** | 22/06/2020 | WT | Unknown | NA | 68 | AstraZeneca |

**Fig. S1**

Summary of donor selection


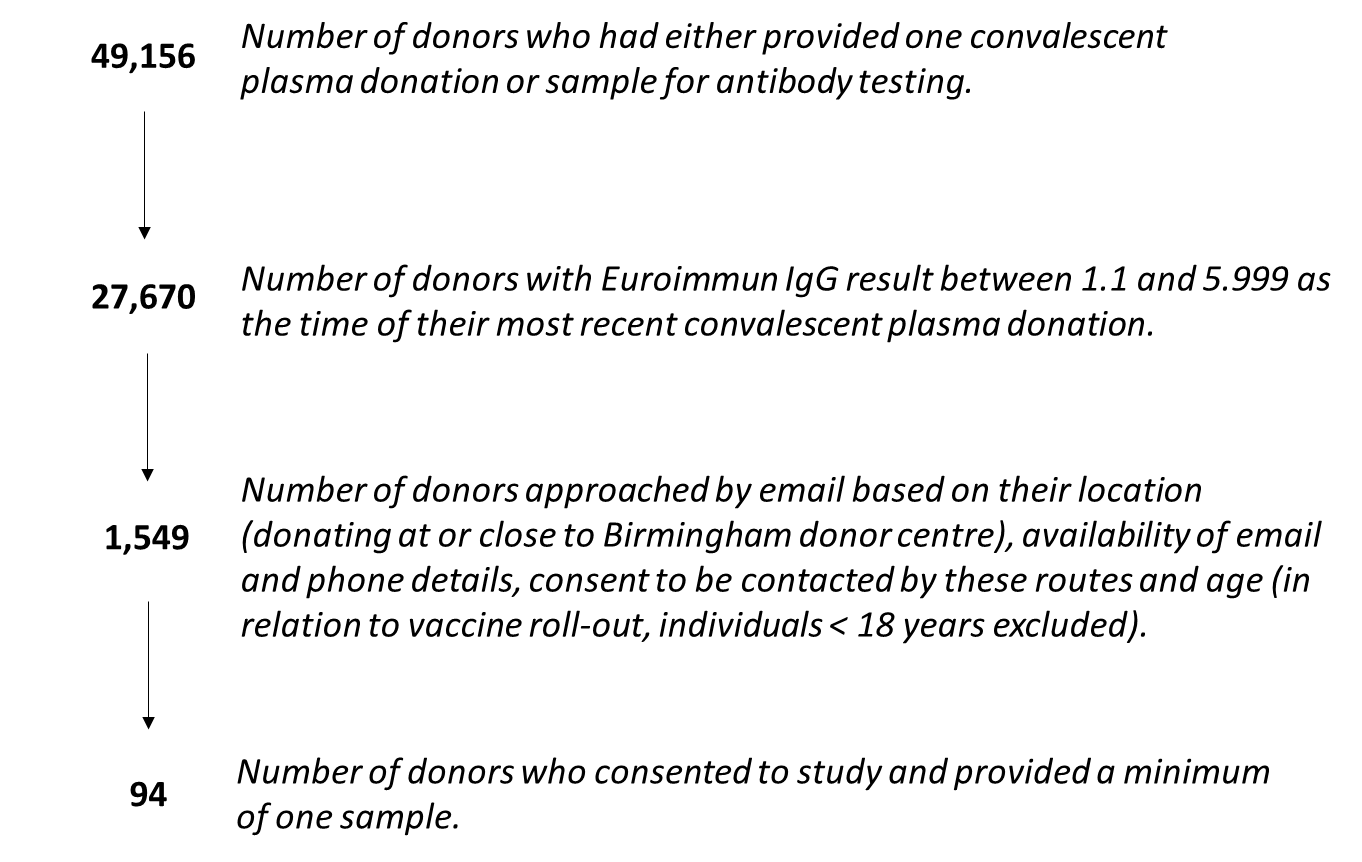


**Fig. S2**.

Comparison of neutralising antibody titres measured by MNA and FRNT.


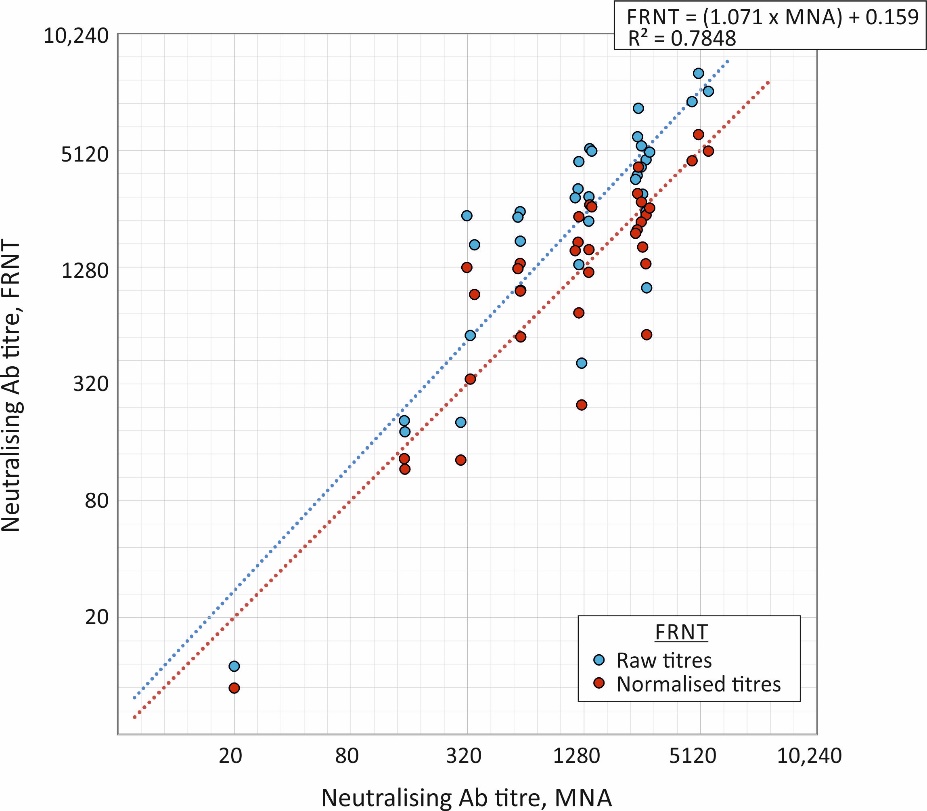


The regression formula derived from the line of best fit of log transformed values was used to normalise FRNT values to those on MNA (replotted as red circles)
